# A qualitative study: Mothers of late preterm infants relate their experiences of community-based care

**DOI:** 10.1371/journal.pone.0174419

**Published:** 2017-03-23

**Authors:** Shahirose S. Premji, Genevieve Currie, Sandra Reilly, Aliyah Dosani, Lynnette May Oliver, Abhay K Lodha, Marilyn Young

**Affiliations:** 1 Faculty of Nursing, University of Calgary, Calgary, Alberta, Canada; 2 Department of Community Health Sciences, Cumming School of Medicine, University of Calgary, Calgary, Alberta, Canada; 3 O’Brien Institute of Public Health, University of Calgary, Calgary, Alberta, Canada; 4 Alberta Children’s Hospital Research Institute, Calgary, Alberta, Canada; 5 School of Nursing and Midwifery, Mount Royal University, Calgary, Alberta, Canada; 6 Department of Paediatrics, Division of Neonatology, Alberta Health Services, Calgary, Alberta, Canada; 7 Prenatal and Postpartum Services, Public Health Calgary Zone, Alberta Health Services, Calgary, Alberta, Canada; Centre Hospitalier Universitaire Vaudois, FRANCE

## Abstract

**Purpose:**

In Alberta, the high occurrence of late preterm infants and early hospital discharge of mother-infant dyads has implications for postpartum care in the community. Shortened hospital stay and complexities surrounding the care of biologically and developmentally immature late preterm infants heighten anxiety and fears. Our descriptive phenomenological study explores mothers’ experience of caring for their late preterm infants in the community.

**Methods:**

Eleven mothers were interviewed using a semi-structured interview guide. Interview transcripts were analysed using an interpretive thematic approach.

**Findings:**

The mothers’ hospital experience informed their perspective that being a late preterm infant was not a “big deal,” and they tended to treat their infant as normal. “Feeding was really problem,” especially the variability in feeding effectiveness, which was not anticipated. Failing to recognize late preterm infants’ feeding distress exemplified lack of knowledge of feeding cues and tendencies to either rationalize or minimize feeding concerns. Public health nurses represent a source of informational support for managing neonatal morbidities associated with being late preterm; however, maternal experiences with public health nurses varied. Some nurses used a directive style that overwhelmed certain mothers. Seeing multiple public health nurses and care providers was not always effective, given inconsistent and contradictory guidance to care. These new and changing situations increased maternal anxiety and stress and influenced maternal confidence in care. Fathers, family, and friends were important sources of emotional support.

**Conclusion:**

After discharge, mothers report their lack of preparation to meet the special needs of their late preterm infants. Current approaches to community-based care can threaten maternal confidence in care. New models and pathways of care for late preterm infants and their families need to be responsive to the spectrum of feeding issues encountered, limit duplication of services, and ensure consistent and effective care that parents will accept.

## Introduction

In Canada, Alberta has the second highest provincial rate (8.7%) of preterm birth reported by hospitals [1]. Late preterm infants (LPIs), born between 34 0/7 and 36 6/7 weeks’ gestational age, make up an increasing proportion (75%) of these preterm infants [2]. Despite their increased short-term morbidities, no significant difference has been reported in length of stay in birth hospital (in days) for LPIs versus term infants, even when considering mode of delivery [3]. Alberta has an average hospital length of stay of 2.5 days for all newborns, lower than the Canadian average of 2.8 days [4].

The immature, inconsistent, and disorganized behaviours that LPIs demonstrate—forgetting to breath, poor state regulation, weak suck and swallow reflex—given their biological and developmental immaturity[5] can create challenges for mothers in interpreting and being responsive to the needs of their newborns [6–8]. Moreover, early hospital discharge places particular demands on the mother to monitor for signs of clinical jaundice and dehydration. In the postpartum period, maternal stress resulting from complexities surrounding care, influences maternal confidence with regards to infant care [9]. Situational circumstances, particularly re-hospitalization for management of morbidities, could increase this maternal stress [10, 11] and anxiety even more [12, 13]. Mothers of preterm infants differ in their adaptation to parenting preterm infants in that they can experience social isolation and lack of support from their partners [14]. Social support can help to alleviate any isolation and lack of home support and also help mothers develop confidence in attending to their infants’ needs [15].

Public health nurses represent an important source of informational and social support, particularly for first time mothers [15]. In Canada, Alberta is the only province that still universally offers early (i.e., 0 to 2 months) community-based care through home or clinic visits by public health nurses within 24 to 48 hours following discharge from hospital, even in instances when the infant remains in the neonatal intensive care unit (NICU). In delivering such care, however, public health nurses follow standards of care for full-term infants with some modifications for LPIs. The subsequent variability in care resulting from adapting empirical knowledge can create a sense of powerlessness accompanied by feelings of great responsibility of care (i.e., moral distress) for the public health nurse [16]. To guide public health nursing interventions, in this exploratory study we examined mothers’ experience of caring for LPIs in the community during the first 2 months following discharge. We addressed the following question: What does it mean to be a mother of a late preterm infant? In the absence of other studies of this kind, we provide information that could enhance care in the community, could alleviate maternal stress, anxiety, and promote optimal mental health of the mother.

## Methods

### Study design

We used an integrated knowledge translation approach in our research. That is, we collaborated with policy decision-makers and clinical managers throughout the research process [17]. As part of a mixed methods design, this descriptive phenomenological inquiry was guided by the conceptual model of Abidin [18] and Leigh and Milgrom [10]. The conceptual model provides a framework for describing the LPIs’ characteristics, situational factors (e.g., monitoring for morbidities), and any imbalance between the needs associated with caring for LPIs and the available perceived resources to meet these needs as contributing factors to maternal stress/anxiety [10, 18]. Details related to this mixed methods study are described elsewhere [19].

### Sample and recruitment

Mothers of LPIs were eligible to participate regardless of mode of delivery and admission status of the LPIs (i.e., newborn nursery, secondary hospital or NICU, or length of stay). Mothers were excluded if they were unable to read/write English or could not be contacted in time for them to complete the maternal confidence in care survey at 3–4 weeks following the birth of their LPIs. As phenomenological studies typically recruit small samples due to larger data sets, only a subset of enrolled mothers participated in interviews. Between April 2013 and June 2014, 72% of enrolled mothers of LPIs in four Calgary hospitals consented to be available for an interview. A multistage purposeful sampling approach, employed over time, was used to reduce the sample to an appropriate number to achieve rich, thick narratives and diversity and breadth of the larger study’s sample [20]. In the first stage of sampling, which occurred at 14–15 weeks, we purposively invited 4–5 mothers who varied with regards to mode of delivery and admission status for interviews. In the second stage that occurred at approximately 23–24 weeks, we sampled 4–5 mothers from those enrolled since the initial stage, for maximum variation to achieve a more heterogeneous sample of mothers (e.g., primiparous and multiparous, infant in newborn nursery and NICU, caesarean section and vaginal deliveries, spontaneous and induced preterm births, and early and late in postpartum period) [20]. In the final stage that occurred at 34–35 weeks, we purposively sampled 4–5 mothers from those enrolled since the last stage for similarity in maternal characteristics, to ensure data saturation which occurs when no new information emerges from the data.[20] When multiple mothers met sampling criterion, we used random sampling (i.e., selecting names out of a box). Eleven of the 14 mothers contacted in the first two attempts participated in an interview.

### Data collection

Data was collected using in-depth, semi-structured interviews (S1 File) that enabled mothers to share rich descriptions and reflect on textural and situational circumstances of caring for their LPIs. Interviews lasted 60–90 minutes in duration. Either of two team members (SP, GC), trained public health practitioners, followed the interview guide to ensure consistency but also exercised discretion to follow up ideas or responses that arose. Briefing and debriefing session identified areas for further exploration in subsequent interviews. Interviews were audio-recorded with permission, transcribed verbatim, reviewed by the respective team member, and supplemented with observational field notes pertaining to parents’ behaviours, mother-infant or father-infant interactions, and infant behaviours. Field notes, recorded after the interview, included descriptions and analysis (e.g., queries, concerns) of unstructured observations made during the conduct of the interview.

The interviewers contacted the mothers and arranged a mutually convenient date and time for the interviews. At the request of one mother, the interview took place by telephone; otherwise, all interviews occurred face-to-face, in the home, and in a private space (LPIs sometimes with the mothers). Mothers received information to facilitate the informed consent process and a $50 grocery food card as reimbursement for the cost of their time. Interviewers disclosed that their employment as public health nurses (casual positions), which promoted reflexivity, opened a richer conversation for interactive experiences permitting meaning-making, and generated data (i.e., field notes) related to situational dynamics between the mothers and their LPIs during feeding. The interviewers reinforced that they would not provide clinical care or counselling during interactions. Data from the PHANTIM database, an administrative database for Postpartum Community Services in Alberta Health Services (AHS) augmented self-reported demographic characteristics.

### Data analysis

We used an interpretive thematic analysis approach [21] that was iterative and involved inductive and deductive reasoning that permitted de-contextualizing and re-contextualizing the data. Two researchers (SP, SR) and a research assistant (LO) independently reviewed the transcripts of each participant manually, and using a template (created by SR) organized significant statements, sentences, or quotes related to key questions and categorized them into clusters of meaning [22]. Organizing and displaying data in this way permitted data reduction, identification of salient themes, and meaning embedded in the language. As a team, we discussed patterns in the codes, then labelled overarching themes and relationships across the narratives of mothers’ interviews with key words or phrases, whenever possible, from the narratives. We noted that our themes reached saturation. When examining relationships to recontextualize data, the team drew from the Abidin [18] framework to assist with explicating the relationships between parenting stress, specifically the parent’s characteristics, child’s characteristics, and situational factors. When there were differences in opinions about patterns, themes, or relationships, the researchers returned to the data. Given budgetary constraints, we did not verify the findings with the mothers.

### Ethics statement

Department heads within AHS formally approved the study during the funding application process. The Conjoint Health Research Ethics Board at the University of Calgary (Ethics ID: E-25040) approved the study. Accordingly, we obtained written informed consent prior to data collection and data access from the AHS Postpartum Community Services’ administrative data sources.

## Results

### Characteristics of participants and birth experience

Tables 1 and 2 detail the characteristics of mothers and infants. The sample included a heterogeneous mix of 11 mothers (e.g., varied gestational ages, parity, and multiple births), 10 of whom filled out a demographic questionnaire. Of the 10 participants who provided data, all were married, 8 had completed a college, trade, or university degree or higher. Eight mothers were born in Canada and 6 were of White/Caucasian ethnicity. Four women had become mothers for the first time. Four of the 10 mothers classified as depressed, had a score of ≥9 on the Edinburgh Postnatal Depression Scale. Two mothers had previous problems with depression and one with alcohol use (10%). Of the 11 participants, 8 had vaginal deliveries. When asked about any health issues at birth, 60% identified their baby had jaundice, while 40% had trouble feeding their child.

**Table 1:** Maternal characteristics.

		Mothers (n = 10)^a^
Characteristics		n
Age, years	Mean (SD)	31.1 (4.0)
Current marital status	Married	10
Highest education level	Some elementary or high school	0
	Graduate high school	1
	Some college, trade, university	1
	Graduated college, trade, university	6
	Some graduate school	1
	Completed graduate school	1
Combined income	$60,000–69,999	3
	$70,000–70,999	1
	$80,000–89,999	1
	$90,000–99,999	1
	$100,000 or more	4
Born in Canada		8
Ethnic background	White/Caucasian	6
	First Nations	1
	Metis	1
	Arab	1
	Chinese	1
Gravid	Primiparous	4
	Multiparous	6
Depression status	Depressed	4
History	Depression	2
	Alcohol use	1

^a^ missing characteristics from 1 mother

**Table 2:** Infant characteristics.

		Babies (n = 12)
Characteristics		n (%)
Gestational age, weeks	34	2 (20%)
	35	2 (20%)
	36	6 (60%)
Birth weight, grams	Mean (SD)	2471.0 (583.3)
	Range	1822–3630
Sex	Boy	6 (50%)
Apgars 1 minute	6	1 (10%)
	7	1 (10%)
	9	9 (90%)
Apgars 5 minutes	9	1 (10%)
	10	10 (100%)
Apgars 10 minutes	10	10 (100%)
Delivery	Vaginally	8 (70%)
	Caesarean section	3 (30%)
Issues on notice of birth	Respiratory issues	1 (10%)
	Cardiovascular issues	0 (0%)
	Hypoglycemia	0 (0%)
	Hypothermia	0 (0%)
	Jaundice	6 (60%)
	Feeding difficulties	4 (40%)
	Sepsis	0 (0%)

### Themes

We present four key themes that emerged from the data: (1) caring for LPIs, (2) feeding the LPI, including challenges and failing to recognize signs of feeding distress, (3) experience with public health nurses, and (4) emotional and instrumental support. These themes were chosen as they provide insight into the mother’s perspectives about what it means to be a mother of a late preterm infant. Another article describes how these experiences impact on their level of stress, confidence in caring for their infants, and reliance on different resources for support.

### 1. Caring for late preterm infants

#### Late preterm infants not a “big deal”

Mothers often reported their happiness in leaving the hospital: “I was definitely, definitely ready to be going home and I knew just being in my own house and just kind of get to our routine” (Mother 10), and “it was more of a relief than anything else…I was in the hospital so much that it was just nice to be home and not have to worry about going back” (Mother 1). Mother 2 recounted that “if it was my first child, it probably would have been totally different experience, but being my second child, I wasn’t as nervous.” Mothers described their transition home as “really great,” saying “when he came home with me, everything was fine” (Mother 4). One mother alternately expressed that “there were a lot of things we didn’t prepare yet” (Mother 9) as she had not anticipated the early birth.

Mothers apparently found little difference between a LPI and a term newborn, and reflected on what shaped this perspective. Explanations or understanding of what they should expect appeared limited as “the information was not there” (Mother 2). When asked if anyone had explained what it means for a baby to be born at 35 weeks, one mother answered, “not at all” (Mother 8). Those who were provided information stated, “they didn’t really give us the impression that it was going to be that big of a deal…may be some feeding issues or something” (Mother 3). One mother reflected, “it’s not really early and they might have some difficulties like jaundice or maybe feeding difficulties” (Mother 9). The “normalization” of their experience was evident even among mothers whose LPIs were in the NICU: “you just feel like a fraud; like, we were in there with this 8 pound tank (laughing) compared to [more fragile infants]…” (Mother 5). Overall, mothers wanted “more information, maybe, initially or about what to expect from a late preterm infant…whether that was like before delivery or post-delivery” (Mother 8), saying that “if there are difference with the preemie things you’ve got to look at and stuff, that would have been nice to know” (Mother 2). Many of the challenges, such as feeding, came as a surprise to these mothers.

### 2. Feeding the late preterm infant

All LPIs faced similar feeding challenges, regardless of feeding method (i.e., breast- or bottle-feeding) or type of hospital stay (i.e., nursery, rooming-in, or NICU). Other interrelated issues such as weight gain and risk of jaundice compounded these hardships.

#### Failing to recognize late preterm infants’ feeding distress

When the interviewers observed feeding interactions, it appeared that mothers failed to recognize feeding cues—tongue thrusting, milk pooling, or dribbling, fingers splaying, and eyes widening—as signs of feeding distress. When probed, mothers labelled these as typical feeding behaviours, and moreover, did not articulate nor demonstrate interventions such as pacing to manage the feeding distress. At the time of the interviews, public health nurses had already assessed mothers and their LPIs, though were not actively following these mothers. A mother who was pleased with her LPI’s feeding as the latch issue had resolved failed to recognize her infant had disorganized behaviour during feeding: “Oh yeah, way better, way better…she knows how to do it now. Like, sometimes she gets frustrated if she’s really hungry but…Oh no, she can latch perfectly now” (Mother 6).

#### “Feeding was really [a] problem”

Mothers described the desire to breastfeed their babies because “she was so little, I wanted to make sure she, she got the best nutrients I could give her” (Mother 10) and because breastfeeding is “work, but it’s worth it… it felt so good to be able to provide something” (Mother 11). Yet, regardless of the method employed, feeding, whether breast- or bottle-feeding, presented problems. Mother 8 explained that her infant was on caffeine; consequently, she was giving him one bottle and during the bottle-feeding, he did “not like the nipple on the bottle. He, um, will gag or tongue thrust and it takes us quite a while to get a few small amount in.” When probed about behaviours during bottle-feeding, another mother also described ineffective bottling such as choking or sputtering: “she still does not, like if you let her…like I have to take it out all the time so she’ll swallow cause otherwise she just like makes swallowing/gulping sound” (Mother 6).

Although mothers identified feeding issues, anatomical and mechanical problems noted in their LPIs were more dominant in the mothers’ voices rather than problems with interpreting feeding cues. Mothers who recognized adverse feeding behaviours attempted to either rationalize or minimize them. For instance, one mother who had observed her LPI pooling milk in the mouth or dribbling milk stated:

I also think it has to do with the fact of the nipple shield, cause like we were using the smallest one first and then I think my milk supply just got really big, and then it was pooling, like milk was just everywhere.

(Mother 3)

A mother who had been counselled in the hospital about feeding problems with LPIs struggled with how her baby appeared so different between feeds, explaining that she could not understand how “in the afternoon you can eat fine, in the evening he’s all (makes choking noise) choking and spitting it up.” Many mothers observed variability in how effectively their LPI feed at different feedings. The pervasive nature of feeding challenges coupled with other interrelated issues increased mothers’ stress and anxiety: “he was losing weight; he wasn’t feeding well; he was jaundiced; I was worried about him” (Mother 9).

Mothers wanted public health nurses to provide more specific information, “especially in the feeding…more geared towards what to expect when a baby has come this early” (Mother 9). The two mothers who identified public health nurses as a social support indicated that it “totally depended on the nurse, I guess” (Mother 3), while the other mother would have liked “even more access to public health” (Mother 8).

### 3. Experience with public health nurses

Public health nurses contacted mothers on the day of or day after returning home from hospital by phone. Mothers received either a home or a clinic visit, and follow-up included repeat visits or a telephone call(s). The duration of follow-up care varied from no repeat contact to weekly follow-ups (varying in frequencies) for 2 weeks.

#### Mixed reviews–“very helpful” versus “very hard”

Mothers reported dissimilar experiences, some very positive and some very negative with different public health nurses. However, most mothers expressed concern about their engagement with the public health nurse. Mothers with a positive perspective were of the view that the public health nurse was “very helpful,” “awesome,” and “wonderful.” One mother felt more reassured because the public health nurse “had the neonatal experience,” so she was “really glad that we got her and her wealth of knowledge” along with “more hands-on experience with the babies and more hand[s]-on experience with feeding and troubles feeding” (Mother 8).

Mothers also described very unsatisfactory experiences with the public health nurses, describing the experience “very hard for me” (Mother 1). One mother felt the public health nurse took over the decision-making: “they feel like they could boss me around and, I’m her mom; ultimately, I know what’s best for her” (Mother 1). Public health nurses who used a non-reassuring approach or directive style in care appeared to cause mothers increased stress or anxiety:

…his temperature was low so I was a little nervous and upset cause I, the public nurse told me if I couldn’t keep the temperature up to go back to the hospital with him. So of course I’m upset and start crying cause, you know.

(Mother 4)

The experience with the public health nurse “wasn’t an overly wonderful experience…I would say it was more her approach” (Mother 10) in the way she conveyed news related to infant issues, which “made me feel like a really bad parent” (Mother 10).

#### Seeing multiple public health nurses was not always effective

Multiple public health nurses and health care professionals provided community-based care to these mothers. One explained, “by the time we got discharged…saw public health [nurses] again. Went to the clinic and saw them [public health nurses] on the Sunday…and we saw our family doctor on the Monday” (Mother 5). For another mother, multiple care providers included “the doctor that delivered her…5 days after she was born…then I saw her paediatrician a week and a bit after she was born” (Mother 1). Some mothers found it “really overwhelming to have a bunch of different nurses come and tell us their own opinion.” (Mother 3). “Each time it was a different [public health nurse]” (Mother 2). Mother 5 had been “in contact with five different public health nurses and heard five different sets of advice that were really contradictory; I had a really hard time trusting what I was being told.” In some instances, this contradictory advice undermined the mother’s confidence:

I kind of lost my ability to…I know how to do this, I know how to feed a baby and I know how to take care of them…I kind of just like lost my gut instinct with so many people telling me so many different things.

(Mother 3)

The same mother wanted more “consistency in care that is being provided and who is providing it” (Mother 3). One mother felt the public health nurse undermined her confidence with regards to her feeding goals for her LPI:

I phoned my family doctor and managed to get in to see him that day…he’s gaining a little bit more slowly…And he said go get a digital weight, you know, and then you track it, call me back if you need to come back in. He’s like, but I’ll follow you up from now on. So he wrote to public health to tell them that he was going to follow me up.

(Mother 5)

### 4. Emotional and instrumental support

Fathers, family, and friends provided important social support to mothers because as Mother 2 reported, “I’m getting really tired.” The fatigue set in because of feeding challenges, travel associated with clinic visits, and care of older children. Fathers provided instrumental support by taking “charge of everything…’cause we just didn’t… [because] you can’t be prepared for this” (Mother 3). Mothers identified fathers as a source of emotional support: “just kind of talking things through with each other…communicating how the day was going” (Mother 11). Family and friends also gave instrumental support:

my two best friends and my sister at the time was out of town, but as soon as she came home, she was here almost every day. My best friend had come and, um, brought me dinner all week when my husband was at work.

(Mother 4)

Mothers spoke highly about the level of support they could acquire from family and friends. A mother said, “we’ve got a huge family support” (Mother 10), and “I would say like just having family support was probably the most helpful thing once we were home” (Mother 8). Some family members were in close vicinity, thus easier to access. For instance, one mother shared, “Yeah, my parents, they like literally live (up the street)—you passed their house to come in.” (Mother 5)

## Discussion

The “normalization” of LPIs appears to set up the mother for stress and distress. Mothers, even those with LPIs in the NICU, “normalized” the birth of their LPI because they had the impression that everything would be fine. Hospital-based health care providers appeared to minimize, in the case of the NICU, the biological and developmental immaturity of the LPIs, thereby perpetuating the mother’s perspective(s). Hospital-based guidelines for care of LPIs emphasize the health care providers’ role in educating parents about neonatal morbidities and early warning signs of clinical deterioration. Unfortunately, this was contrary to some health care providers in the hospital who have given the mother the impression that “everything will be fine.” Although mothers felt ready to transition home, they were not completely sure what it meant to care for a “small” or “early” baby at home or in the community. Our findings suggest issues with either knowledge translation (i.e., application of evidence-informed guidelines) by hospital health care providers or knowledge uptake by parents.

The “normalization” of their LPIs, by both the mothers and health care providers, contributed to their inability to recognize or appreciate signs of distress during feeding. Public health nurses have the opportunity to point out to mothers the complexity of their LPIs by drawing the mothers’ attention to feeding difficulties and jaundice. It appeared that some public health nurses were overly cautious and hyper-responsive to an infant’s condition adding to maternal stress, which occasionally set up a conflictual relationship between the mother and public health nurse. Some mothers found their interactions with the public health nurse “very hard” given their sense of urgency in attending to the feeding problems and jaundice. At times, the directive communication style of individual public health nurses discredited the work of public health nurse as a whole.

A qualitative study employing a grounded theory method to describe the 6–8 weeks breast-feeding experience of 10 mothers of LPIs also found feeding to be a tenuous process of trial and error, accompanied with uncertainty and extensive effort [23]. Mothers, it appears, required more education to consider visible signs of feeding effectiveness such as milk transfer and infant satiety when appraising “worth against uncertain work” [23]. Similarly, such notions of breast-feeding a “normal” healthy infant caused significant angst for mothers in our study because they could not apparently understand why their healthy infants behaved differently. Maternal distress among mothers’ of LPIs is associated with their breastfeeding performance (or lack thereof), and mothers perspectives related to the behaviour of her infant [24, 25]. LPIs have increased risk of cognitive, emotional, and behavioural problems than term infants in the first 7 years of life [26, 27]. The mothers’ inability to be responsive to her LPI during feeding has significant implications, specifically given the frequency of these interactions (10–12 feeding per day), on infant attachment security and later development [28] [29]. In short, the cumulative nature of this stress can have negative consequences for the LPIs’ growth and development [30].

With respect to social support, fathers were a particularly important source of emotional and instrumental support for mothers. Mothers also drew this level of support from family and friends. In some cases when the experience with the public health nurse was positive, the experience provided another source of social support from the public health nurse. However, other instances were not positive as the individual public health nurses’ approach to care resulted in mothers’ feeling like they did not know what they were doing and they felt incompetent. Mothers need information and a therapeutic approach that fosters confidence in care.

In Alberta, public health nurses use modified standards of care for LPIs, that is, standards developed for term infants and adapted for application for LPIs as well as experiential learning (e.g., NICU) to guide care practices. Lack of evidence-informed public health nursing practice guidelines or standards of care specifically for LPIs can explain inconsistencies in the provision of care to mothers and their LPIs. The increase in health care utilization observed among LPIs, particularly in the first 30 days following discharge from the hospital [31–33] may be due in part to incongruences between expected and observed infant characteristics, inconsistencies in care between public health nurses, and heightened concerns related to morbidities associated with being a LPI. An integrated care approach with well-defined standards and pathways of care specifically for LPIs can limit duplication of services (i.e., multiple care providers monitoring the same challenges), ensure consistency in care, enable the right care provider to deliver service at the right time and in the right place, and reduce maternal anxiety, stress, and fatigue.

Our participants’ willingness to be interviewed suggests that they may be interested in collaborating to inform improvements in postpartum community services. A participatory approach would assure that mothers contribute to the research question(s), identify core public health competencies and interventions, as well as specify outcomes responsive to their needs. In the long-term such an approach would hold the promise that mothers of LPIs would make use of the new knowledge to improve their health and well-being and that of their LPIs. The understanding gained from this study has enabled the researchers to generate hypotheses that will inform future studies and ensure judicious use of resources. For example, practical information and a feeding toolkit for mothers of LPIs may enable them to recognize and be responsive to feeding cues, thereby increase maternal confidence in care and reduce maternal anxiety.

## Conclusions

Mothers in our study seem unprepared for the special needs of their LPI, and current approaches to hospital and community based care require improvement. Mothers prefer community-based care approaches aligned with their perspectives or expectation(s) of care for their LPI. Mothers of LPIs want an integrated system of care responsive to the spectrum of complexities of their LPI including consistent, effective, and acceptable care practices that promote a more positive experience for mothers caring for their LPIs in the community.

## Supporting information

S1 File Appendix (DOCX)
