# Supplementary material for: A qualitative study: Mothers of late preterm infants relate their experiences of community-based care
Source: PLoS One. 2017 Mar 23;12(3):e0174419. doi: 10.1371/journal.pone.0174419 (PMC5363959; doi:10.1371/journal.pone.0174419)
Supplement: S1 File — (DOCX) [file pone.0174419.s001.docx]

**Appendix 1. Interview Guide for Mothers’ Experience**

Can you tell me more about what it was like when you were told your baby was a late preterm infant? What was that like for you?

Describe your experience of caring for your baby since coming home?

- When thinking back on the initial days you were home after discharge from hospital, what do you remember about those days? (For those who have been home for a couple of weeks).
- Tell me about the last feeding of your baby. What was that like for you?
- Tell me about the moments you feel confident about caring for your baby (i.e., looking after your late preterm infant).
- Tell me about the challenges in caring for your baby?
- How did you cope? Who or what helped you during these periods?
- What other resources would have proved helpful during these periods?

Have you met with the public health nurse since coming home from the hospital? What was that experience like for you.

How do you feel about your wife/partner’s participation in care?
